# An eleven metabolic gene signature-based prognostic model for clear cell renal cell carcinoma

**DOI:** 10.18632/aging.104088

**Published:** 2020-11-18

**Authors:** Yue Wu, Xian Wei, Huan Feng, Bintao Hu, Bo Liu, Yang Luan, Yajun Ruan, Xiaming Liu, Zhuo Liu, Shaogang Wang, Jihong Liu, Tao Wang

**Affiliations:** 1 Department of Urology, Tongji Hospital, Tongji Medical College, Huazhong University of Science and Technology, Wuhan 430030, Hubei, China; 2 Institute of Urology, Tongji Hospital, Tongji Medical College, Huazhong University of Science and Technology, Wuhan 430030, Hubei, China; 3 Department of Oncology, Tongji Hospital, Tongji Medical College, Huazhong University of Science and Technology, Wuhan 430030, Hubei, China

**Keywords:** clear cell renal cell carcinoma, metabolic genes, prognostic model, bioinformatics

## Abstract

In this study, we performed bioinformatics and statistical analyses to investigate the prognostic significance of metabolic genes in clear cell renal cell carcinoma (ccRCC) using the transcriptome data of 539 ccRCC and 72 normal renal tissues from TCGA database. We identified 79 upregulated and 45 downregulated (n=124) metabolic genes in ccRCC tissues. Eleven prognostic metabolic genes (*NOS1, ALAD, ALDH3B2, ACADM, ITPKA, IMPDH1, SCD5, FADS2, ACHE, CA4, and HK3*) were identified by further analysis. We then constructed an 11-metabolic gene signature-based prognostic risk score model and classified ccRCC patients into high- and low-risk groups. Overall survival (OS) among the high-risk ccRCC patients was significantly shorter than among the low-risk ccRCC patients. Receiver operating characteristic (ROC) curve analysis of the prognostic risk score model showed that the areas under the ROC curve for the 1-, 3-, and 5-year OS were 0.810, 0.738, and 0.771, respectively. Thus, our prognostic model showed favorable predictive power in the TCGA and E-MTAB-1980 ccRCC patient cohorts. We also established a nomogram based on these eleven metabolic genes and validated internally in the TCGA cohort, showing an accurate prediction for prognosis in ccRCC.

## INTRODUCTION

Clear cell renal cell carcinoma (ccRCC) is the most common among all renal cell carcinoma (RCC) subtypes, and accounts for 75% of all renal tumors [1]. It represents a hypervascular parenchymal malignancy originating from the proximal tubular cells of the nephron [2]. The prognosis of patients with metastatic ccRCC is poor and the five-year survival rate is less than 10% [3, 4]. In the last two decades, the roles of several genes in the regulation of the growth and progression of ccRCC have been recognized, but, the underlying pathogenetic mechanisms are still not understood fully.

Metabolic reprogramming is a common feature of tumor cells and is associated with tumor growth and progression [5]. The most common metabolic changes observed in several types of cancer cells include upregulation of nucleotide biosynthesis and downregulation of mitochondrial metabolism [6]. Peng et al. investigated the mRNA expression patterns of seven major metabolic processes in the tumor subtypes of 33 different cancers and found that those with increased carbohydrate, nucleotide, and vitamin/co-factor metabolism were consistently associated with worse prognosis [7]. Aberrant regulation of the tricarboxylic acid (TCA) cycle, glutamine metabolism, and lipid metabolism are associated with the growth and progression of RCC [8–11]. Hakimi et al. performed metabolomic profiling of ccRCC tissues and found significant alterations in central carbon metabolism, one-carbon metabolism, and the antioxidant response; metabolic changes in glutathione and cysteine/methionine metabolism pathways were associated with progression and metastasis of ccRCC; a metabolic differentiation group in chromophobe RCC was associated with worse survival outcomes [12]. The investigation related to ccRCC have mainly focused on the metabolic changes involved ccRCC, but, the information regarding the expression patterns of key metabolism-related genes involved in the metabolic reprogramming and the metabolomic characteristics of ccRCC are not clear.

Hence, in the current study, we systematically analyzed the transcriptome data from ccRCC patient tissues to identify the metabolic genes that can accurately predict the prognosis of ccRCC patients.

## RESULTS

### Identification of differentially expressed metabolic genes in the ccRCC patients

**Figure 1:**
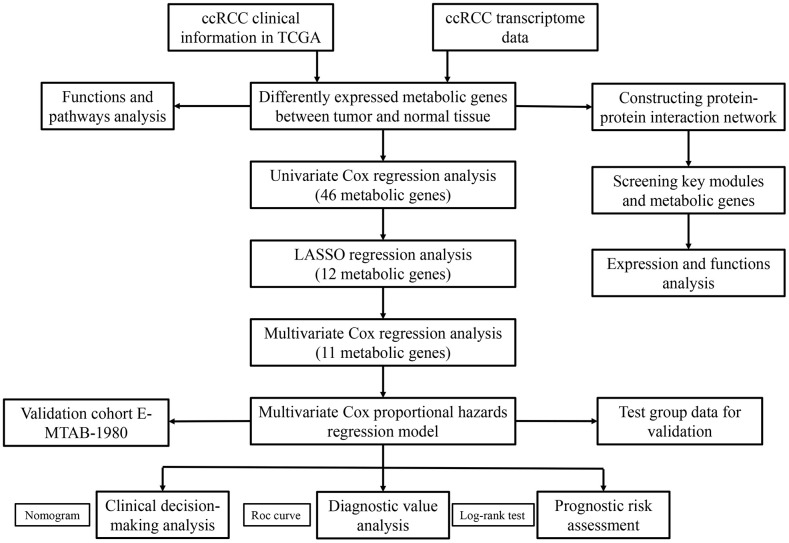
**The flow chart of the study strategy for identifying metabolic genes with prognostic significance in ccRCC.**

**Figure 2:**
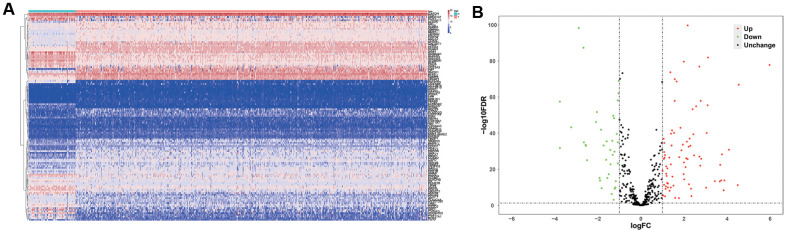
**Differentially expressed metabolic genes in ccRCC samples.** (**A**) The heat map shows the expression of 124 differentially expressed metabolic genes in ccRCC and normal renal tissue samples. (**B**) The volcano plot shows the upregulated or downregulated metabolic genes in the ccRCC samples relative to normal renal tissue samples.

Figure 1 shows the study strategy used to systematically analyze the prognostic prediction values of the metabolic genes in ccRCC. We downloaded the ccRCC transcriptome data for 72 normal renal tissue samples and 539 ccRCC tissue samples from The Cancer Genome Atlas (TCGA) database. Then, we identified differentially expressed metabolic genes in ccRCC using the edgeR package (http://www.bioconductor.org/packages/release/bioc/html/edgeR.html) by screening the 1,466 metabolism-related genes that are listed in the 70 metabolism-related gene sets from the Kyoto Encyclopedia of Genes and Genomes (KEGG) database in the gene set enrichment analysis (GSEA) website (https://www.gsea-msigdb.org/gsea/msigdb/collections.jsp#C2). We used the selection criteria as |log_2_ fold change (FC)| >1.0 and *P* < 0.05 and identified 124 differentially expressed metabolic genes, including 79 up-regulated and 45 down-regulated genes (Figure 2).

### Functional enrichment analysis of the differentially expressed metabolic genes

Next, we performed Gene Ontology (GO) and KEGG functional enrichment analysis of the 124 differentially expressed metabolic genes using the WEB-based Gene Set Analysis Toolkit (WebGestalt) online tool and the results are shown in Table 1. The significantly enriched biological processes were small molecule catabolism, organic acid biosynthesis, organic hydroxy compound metabolism, cellular amino acid metabolism, generation of precursor metabolites and energy, fatty acid derivative metabolism, monosaccharide metabolism, nucleoside phosphate biosynthesis, ribonucleotide metabolism, and fatty acid metabolism. The significantly enriched cellular components were the mitochondrial matrix, ficolin-1-rich granule, and the myelin sheath. The enriched molecular functions were the co-factor binding, oxidoreductase activity acting on paired donors with incorporation or reduction of molecular oxygen, lyase activity, organic acid binding, iron ion binding, monooxygenase activity, transferase activity, transferring glycosyl groups, vitamin binding, oxidoreductase activity acting on the aldehyde or oxo group of donors, and oxidoreductase activity acting on CH-OH group of donors. KEGG analysis showed enrichment in metabolic pathways including those involved in drug metabolism, retinol metabolism, chemical carcinogenesis, purine metabolism, porphyrin and chlorophyll metabolism, steroid hormone biosynthesis, metabolism of xenobiotics by cytochrome P450, and carbon metabolism.

**Table 1:** KEGG pathway and GO enrichment analysis of differentially expressed metabolic genes.

	**GO term**	***P*-value**	**FDR**
Biological processes	small molecule catabolic process	0	0
	organic acid biosynthetic process	6.55E-14	2.78E-11
	organic hydroxy compound metabolic process	3.20E-10	9.06E-8
	cellular amino acid metabolic process	4.93E-10	1.05E-7
	generation of precursor metabolites and energy	6.84E-10	1.16E-7
	fatty acid derivative metabolic process	3.62E-9	5.12E-7
	monosaccharide metabolic process	5.46E-9	6.63E-7
	nucleoside phosphate biosynthetic process	1.17E-8	0.000001
	ribonucleotide metabolic process	1.26E-8	0.000001
	fatty acid metabolic process	2.06E-8	0.000002
Cellular component	mitochondrial matrix	0.000070	0.012090
	ficolin-1-rich granule	0.000265	0.022800
	myelin sheath	0.000774	0.044374
Molecular function	cofactor binding	6.66E-16	1.88E-13
	oxidoreductase activity, acting on paired donors, with incorporation or reduction of molecular oxygen	7.64E-14	1.08E-11
	lyase activity	1.11E-11	1.04E-9
	organic acid binding	9.99E-9	6.93E-7
	iron ion binding	1.23E-8	6.93E-7
	monooxygenase activity	1.75E-8	8.24E-7
	transferase activity, transferring glycosyl groups	4.15E-8	0.000002
	vitamin binding	5.28E-8	0.000002
	oxidoreductase activity, acting on the aldehyde or oxo group of donors	0.000003	0.000084
	oxidoreductase activity, acting on CH-OH group of donors	0.000003	0.000095
KEGG pathway	metabolic pathways	0	0
	drug metabolism	2.43E-12	3.71E-10
	retinol metabolism	3.41E-12	3.71E-10
	chemical carcinogenesis	6.20E-11	3.62E-9
	purine metabolism	6.67E-11	3.62E-9
	porphyrin and chlorophyll metabolism	1.24E-9	5.76E-8
	steroid hormone biosynthesis	3.52E-9	1.43E-7
	metabolism of xenobiotics by cytochrome P450	3.97E-9	1.44E-7
	carbon metabolism	6.20E-8	0.000002

### Protein-protein interaction (PPI) network and key co-expression modules

**Figure 3:**
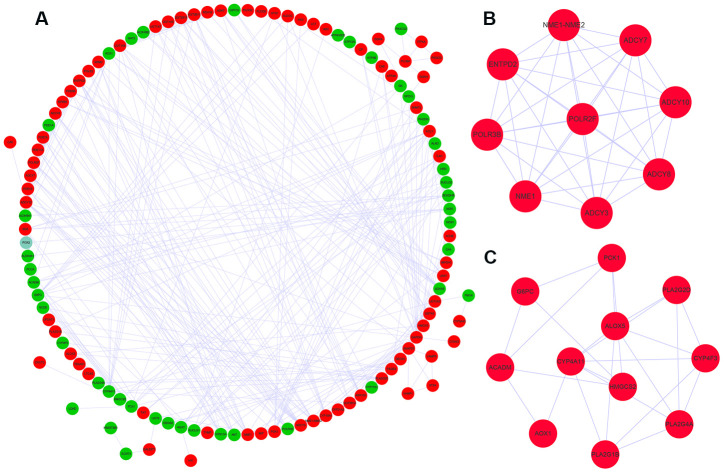
**Protein-protein interaction network and key co-expression modules.** (**A**) The protein-protein interaction (PPI) network shows the interactions between 124 differentially expressed metabolic genes. (**B**, **C**) The two key modules consisting of co-expressing differentially expressed metabolic genes, module 1 and module 2 are shown. The red and green circles denote upregulated and downregulated metabolic genes, respectively.

We used the STRING database and the cytoscape software to construct a PPI network of the differently expressed metabolic genes in ccRCC tissues. The PPI network consisted of 381 edges and 118 nodes (Figure 3A). We also identified two key co-expression modules using the Molecular Complex Detection (MCODE) plug-in of the Cytoscape software. Module 1 consisted of 36 edges and 9 nodes (Figure 3B), and module 2 consisted of 24 edges and 11 nodes (Figure 3C).

### ROC curve analysis of the hub metabolic genes

**Figure 4:**
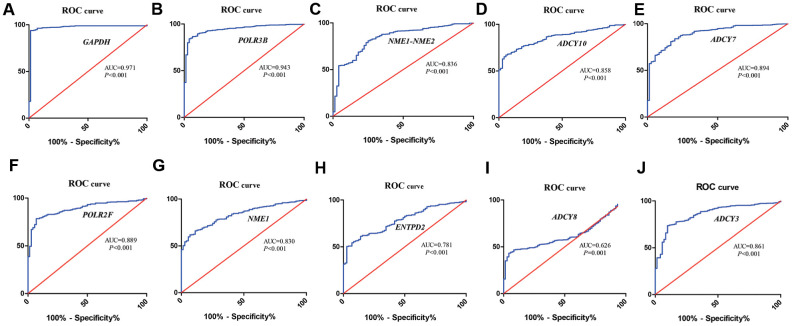
**ROC curve analysis of hub metabolic genes.** The figure shows the ROC curves evaluating the diagnostic accuracy of the 10 hub metabolic genes, namely, (**A**) *GAPDH*; (**B**) *POLR3B*; (**C**) *NME1-NME2*; (**D**) *ADCY10*; (**E**) *ADCY7*; (**F**) *POLR2F*; (**G**) *NME1*; (**H**) *ENTPD2;* (**I**) *ADCY8*; (**J**) *ADCY3* in ccRCC patients.

We identified ten metabolic genes as hub genes using the cytoHubba plug-in of the Cytoscape software and the maximal clique centrality (MCC) algorithm. We then performed the receiver operating characteristic (ROC) curve analysis to evaluate the efficacy of these ten hub genes to discriminate between tumor and normal renal tissue. As shown in Figure 4, the area under the ROC curve (AUC) values showed good diagnostic accuracy, namely, GAPDH (AUC=0.971, *P*<0.001), POLR3B (AUC=0.943, *P*<0.001), NME1-NME2 (AUC=0.836, *P*<0.001), ADCY10 (AUC=0.858, *P*<0.001), ADCY7 (AUC=0.894, *P*<0.001), POLR2F (AUC=0.889, *P*<0.001), NME1 (AUC=0.830, *P*<0.001), ENTPD2 (AUC=0.781, *P*<0.001), ADCY8(AUC=0.626, *P*=0.001), ADCY3 (AUC=0.861, *P*<0.001).

### Analysis of mutation frequencies and copy number variations of the hub genes

**Figure 5:**
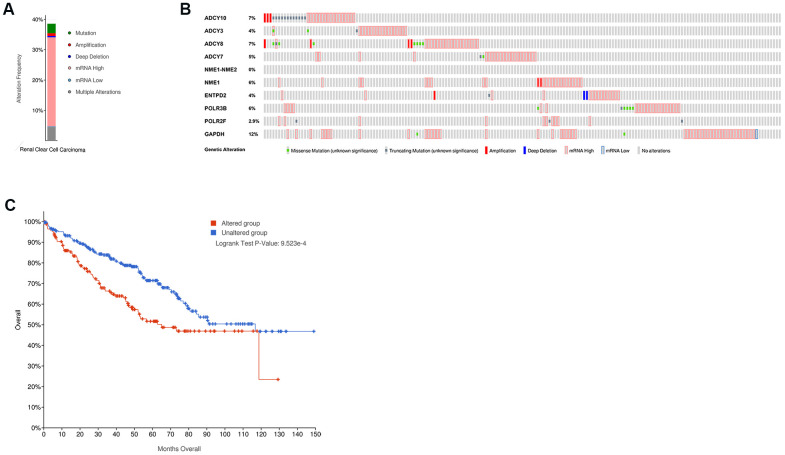
**The mutation frequency of the ten hub metabolic genes in the ccRCC patients (TCGA, Firehose Legacy).** (**A**) The overall mutation frequency of the hub metabolic genes in 446 ccRCC patients. (**B**) The mutation frequency of the individual hub metabolic genes in 446 ccRCC patients. (**C**) Kaplan–Meier survival curves show the OS of ccRCC patients with mutations in the hub metabolic genes (n=172) compared to those without mutations in the hub metabolic genes (n=274).

We used the cBioPortal online tool to analyze the mutation frequencies in the 10 hub genes, namely, GAPDH, POLR3B, NME1-NME2, ADCY10, ADCY7, POLR2F, NME1, ENTPD2, ADCY8, and ADCY3 in ccRCC tissues (TCGA, Firehose Legacy). We observed mutations in these 10 candidate hub genes in 39% (172 of 446) of the ccRCC patients (Figure 5A). The mutation frequency in each of the ten candidate hub genes ranged from 0% to 12% (Figure 5B). Kaplan– Meier survival curve analysis showed that the overall survival (OS) was significantly shorter in the ccRCC patients with mutations in the candidate hub genes compared to the ccRCC patients without mutations in the candidate hub genes (Figure 5C).

### Identification of the prognosis-related metabolic genes

We used the caret R package to divide the ccRCC samples (n=539) from TCGA database into the training group (n=381) and the test group (n=158). We first investigated the prognostic significance of the 124 differentially expressed metabolic genes in the training group using univariate Cox regression analysis and identified 46 potential candidate metabolic genes (Supplementary Table 1). Then, we performed the least absolute shrinkage and selection operator (LASSO) regression analysis and identified 12 metabolic genes with potential prognostic value, including NOS1, ALAD, ALDH3B2, ACADM, ITPKA, IMPDH1, SCD5, FADS2, ACHE, CA4, HK3, and VAMP1 (Supplementary Figure 1). Multivariate Cox regression analysis showed that 11 of the 12 genes, namely, NOS1, ALAD, ALDH3B2, ACADM, ITPKA, IMPDH1, SCD5, FADS2, ACHE, CA4, and HK3 independently predicted prognosis of ccRCC patients.

### Evaluation of the 11-metabolic gene signature-based prognostic risk score model

We performed stepwise Cox regression analysis using the eleven metabolic genes to construct a prediction model (Table 2). The risk score of each patient was calculated with the following formula: *Risk score* = (-0.0555 * Exp NOS1) + (0.0180 * Exp ALAD) + (0.1302 * Exp ALDH3B2) + (-0.0948 * Exp ACADM) + (0.0490 * Exp ITPKA) + (0.1788 * Exp IMPDH1) + (-0.1354 * Exp SCD5) + (0.1488 * Exp FADS2) + (0.0594 * Exp ACHE) + (-0.0570 * Exp CA4) + (0.2213 * Exp HK3).

**Table 2:** Multivariate Cox regression analysis to identify prognosis-related metabolic genes.

**Gene**	**Coef**	**Exp(coef)**	**se(coef)**	**z**	***Pr* (>|z|)**
NOS1	-0.0555	0.9460	0.0694	-0.8002	0.4236
ALAD	0.0180	1.0181	0.2081	0.0863	0.9312
ALDH3B2	0.1302	1.1391	0.0538	2.4218	0.0154
ACADM	-0.0948	0.9095	0.1425	-0.6653	0.5058
ITPKA	0.0490	1.0502	0.0583	0.8396	0.4011
IMPDH1	0.1788	1.1958	0.2212	0.8084	0.4189
SCD5	-0.1354	0.8734	0.0687	-1.9707	0.0488
FADS2	0.1488	1.1604	0.0890	1.6715	0.0946
ACHE	0.0594	1.0612	0.0646	0.9189	0.3582
CA4	-0.0570	0.9446	0.0577	-0.9884	0.3230
HK3	0.2213	1.2477	0.1045	2.1166	0.0343

**Figure 6:**
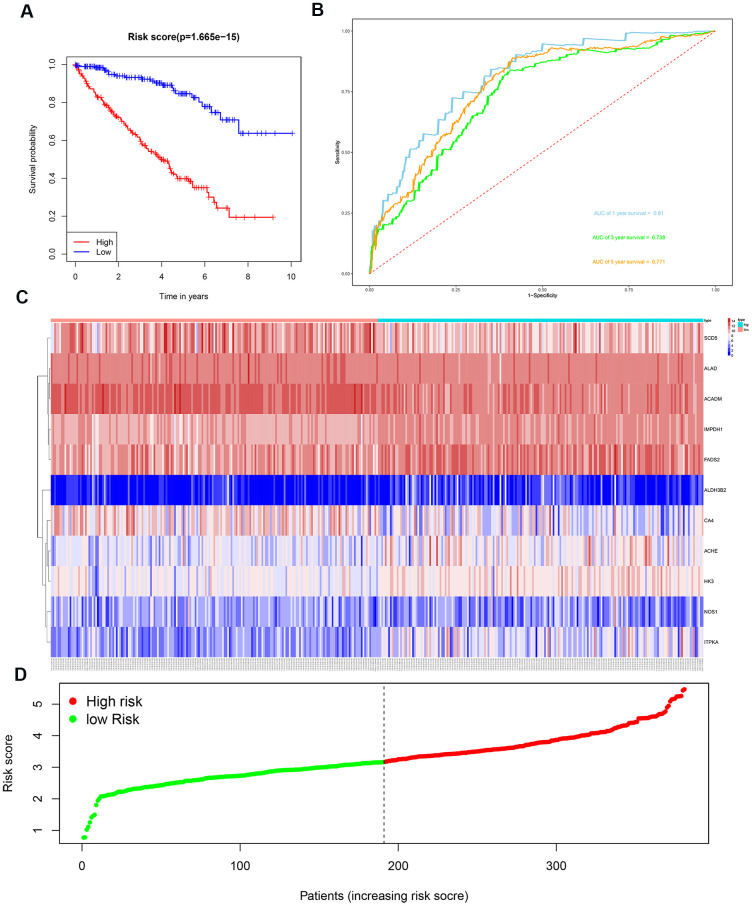
**Risk score analysis of the 11 metabolic gene signature-based prognostic model in the training group ccRCC patients.** (**A**) Kaplan-Meier survival curve analysis shows the overall survival of high- (n=190) and low-risk (n=191) training group ccRCC patients based on the median risk score calculated using the 11 metabolic genes-based prognostic model. (**B**) Time dependent ROC curve analysis shows the prognostic performance of the 11-metabolic gene signature-based prognostic model in predicting 1-year, 3-year, and 5-year survival times of the high- and low-risk training group ccRCC patients. (**C**) Heat map shows the expression of the 11 metabolic genes in high- and low-risk training group ccRCC patients. (**D**) Risk curve analysis of the 11 metabolic genes in high- and low-risk training group ccRCC patients.

**Figure 7:**
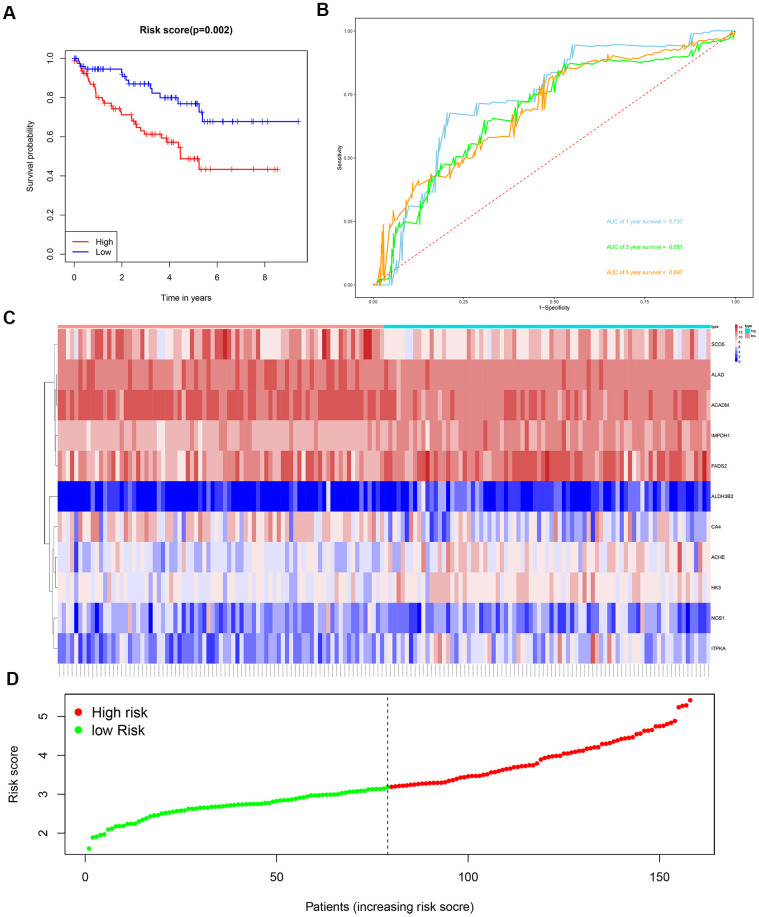
**Risk score analysis of the 11 metabolic gene signature-based prognostic model in the test group ccRCC patients.** (**A**) Kaplan-Meier survival curve analysis shows the overall survival of high- (n=79) and low-risk (n=79) test group ccRCC patients based on the median risk score calculated using the 11 metabolic gene signature-based prognostic model. (**B**) Time dependent ROC curve analysis shows the prognostic performance of the 11 metabolic gene signature-based prognostic model in predicting 1-year, 3-year, and 5-year survival times of the high- and low-risk test group ccRCC patients. (**C**) Heat map shows the expression of the 11 metabolic genes in high- and low-risk test group ccRCC patients. (**D**) Risk curve analysis of the 11 metabolic genes in high- and low-risk test group ccRCC patients.

**Figure 8:**
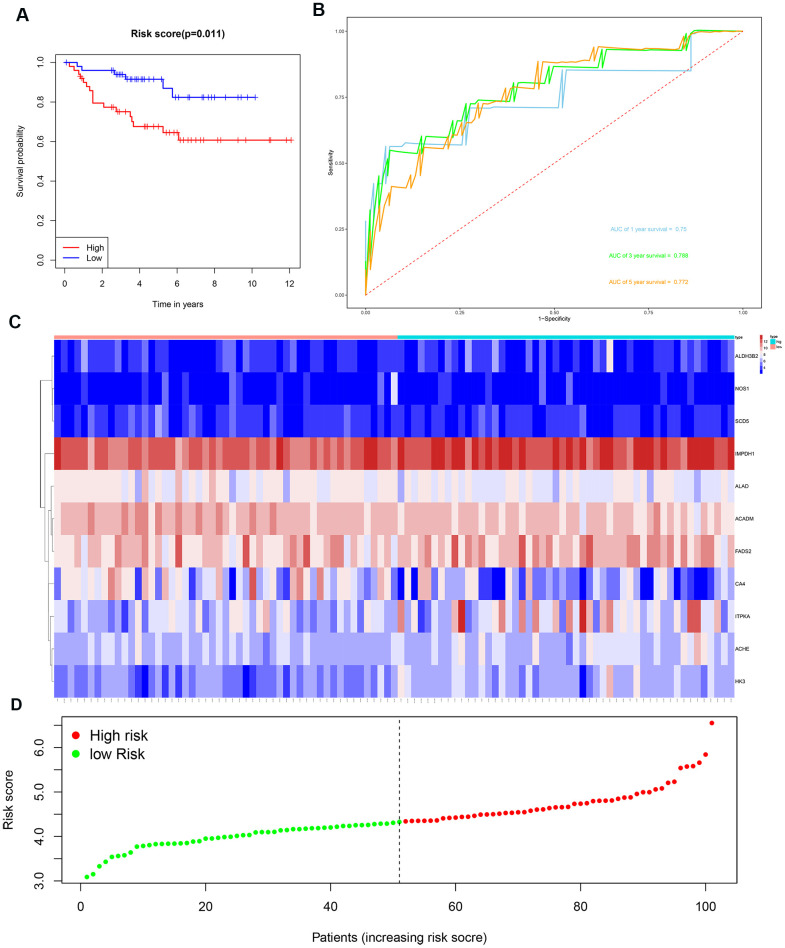
**Risk score analysis of the 11 metabolic gene signature-related prognostic model in the E-MTAB-1980 cohort.** (**A**) Kaplan-Meier survival curve analysis shows the overall survival of high- (n=50) and low-risk (n=51) ccRCC patients from the E-MTAB-1980 cohort based on the median risk score calculated using the 11 metabolic gene signature-based prognostic model. (**B**) Time dependent ROC curve analysis shows the prognostic performance of the 11 metabolic gene signature-based prognostic model in predicting 1-year, 3-year, and 5-year survival times of the ccRCC patients from the E-MTAB-1980 cohort. (**C**) Heat map shows the expression of the 11 metabolic genes in high- and low-risk ccRCC patients from the E-MTAB-1980 cohort. (**D**) Risk curve analysis of the 11 metabolic genes in high- and low-risk ccRCC patients from the E-MTAB-1980 cohort.

We then divided the 381 patients in the training group into high- and low-risk groups based on the median risk score. Kaplan-Meier survival curve analysis showed that the OS was significantly shorter for the high-risk group ccRCC patients compared to the low-risk group ccRCC patients (Figure 6A). Time-dependent ROC analysis of the 11-metabolic gene signature-based prognostic risk score model showed that AUC values for 1-year, 3-year and 5-year OS were 0.810, 0.738, and 0.771, respectively (Figure 6B). Figures 6C, 6D show the heat map and risk curve analyses of the eleven genes in the high- and low-risk group ccRCC patients, respectively. These results show moderate performance of the 11-metabolic gene signature-based prognostic prediction model. We then calculated the risk scores of the test group patients using the same prognostic risk score formula and assessed the predictive performance of the prognostic risk model. Kaplan-Meier survival curve analysis showed that the OS was significantly shorter for the ccRCC patients in the high-risk group compared to the low-risk group ccRCC patients (Figure 7). We obtained similar results for the E-MTAB-1980 cohort (Figure 8). These results demonstrate stable performance of the 11-metabolic gene signature-based prognostic prediction model.

**Figure 9:**
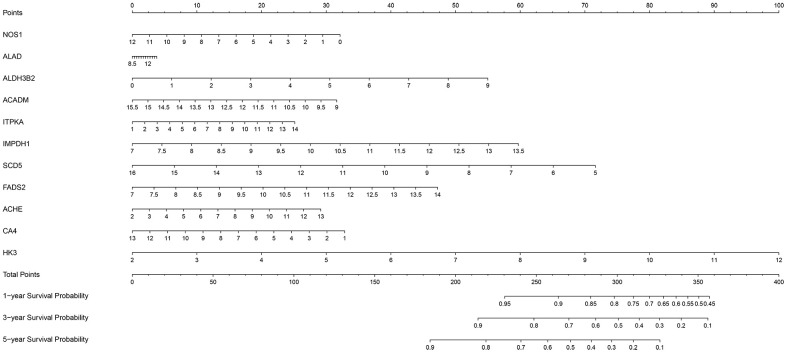
**The nomogram based on the 11 metabolic genes for predicting the one- year, three-year and five-year OS of ccRCC patients.**

We then constructed a nomogram based on the 11-metabolic gene signature to establish a quantitative method for ccRCC prognosis (Figure 9). We calculated total points for each ccRCC patient by adding up the points for each variable and normalized it to a distribution between 0 and 100. Then, we estimated the one-year, three-year and five-year survival rates for each of the ccRCC patient’s by drawing a line perpendicular to the prognosis axis and the total point’s axis. We then performed Cox regression analysis and observed that age, tumor grade, tumor stage, primary tumor location, lymph node infiltration, distant metastasis and the prognostic risk score were significantly associated with the OS of ccRCC patients (Table 3). Multiple regression analysis showed that age (*P=*0.003), tumor grade (*P=*0.016), tumor stage (*P*<0.001), primary tumor location (*P*=0.038) and the prognostic risk score (*P* < 0.001) were independent prognostic factors associated with OS (Table 3).

**Table 3:** The prognostic value of different clinical parameters.

	**HR**	**Univariate analysis**	***P*-value**		**HR**	**Multivariate analysis**	***P*-value**
		**95%CI**				**95%CI**	
Age	2.03	1.40-2.93	<0.001		1.76	1.21-2.58	0.003
Gender	0.89	0.61-1.28	0.5231		0.86	0.59-1.26	0.429
Grade	2.55	1.99-3.27	<0.001		1.41	1.07-1.88	0.016
Stage	2.03	1.73-2.38	<0.001		2.26	1.59-3.21	<0.001
T	2.04	1.68-2.48	<0.001		0.68	0.47-0.98	0.038
N	0.83	0.69-0.99	0.0438		0.87	0.72-1.05	0.149
M	1.85	1.41-2.43	<0.001		0.74	0.44-1.23	0.241
Risk score	2.72	2.16-3.42	<0.001		1.88	1.41-2.51	<0.001

HR, hazard ratio; CI, confidence interval.

**Figure 10:**
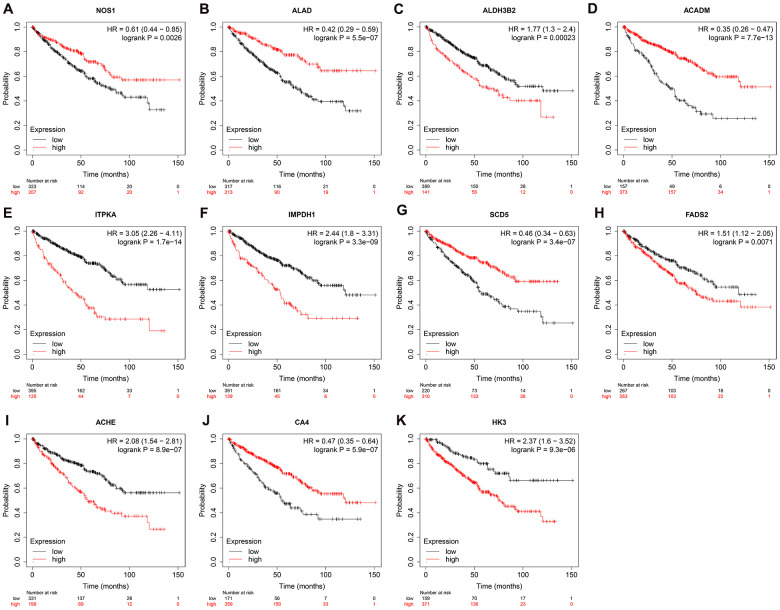
**Prognostic value of the prognosis related metabolic genes in ccRCC by Kaplan-Meier plotter.** Survival curve analysis of ccRCC patients based on the expression status of (**A**) NOS1; (**B**) ALAD; (**C**) ALDH3B2; (**D**) ACADM; (**E**) ITPKA; (**F**) IMPDH1; (**G**) SCD5; (**H**) FADS2; (**I**) ACHE; (**J**) CA4; (**K**) HK3 genes.

**Figure 11:**
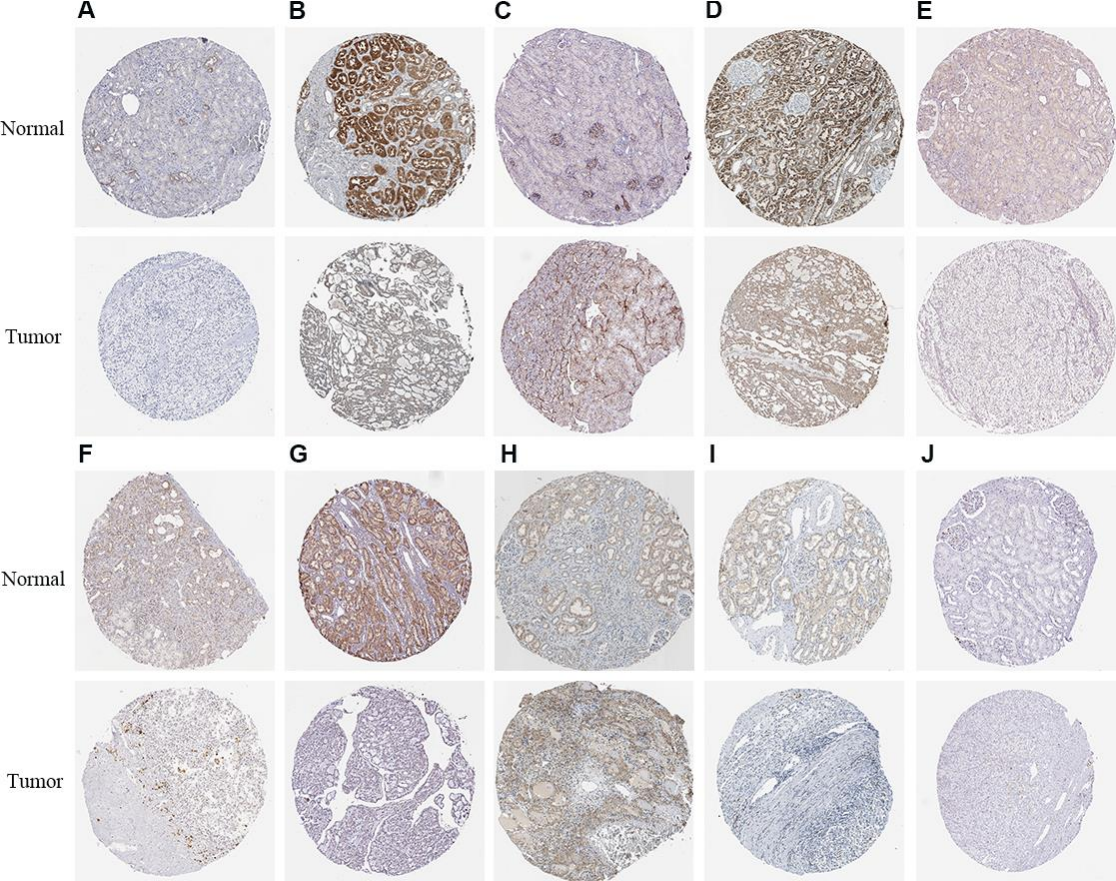
**The expression status of the prognosis related metabolic proteins in ccRCC and normal renal tissues in the HPA database.** (**A**) NOS1; (**B**) ALAD; (**C**) ALDH3B2; (**D**) ACADM; (**E**) ITPKA; (**F**) IMPDH1; (**G**) SCD5; (**H**) FADS2; (**I**) CA4; (**J**) HK3.

We used Kaplan–Meier plotter (http://www.proteinatlas.org/) online tool to determine the relationship between these metabolic genes and OS, and survival curve analysis showed that all the eleven metabolic genes were associated with the OS in the ccRCC patients (Figure 10). We used the immunohistochemical-stained results from the Human Protein Atlas database (https://www.proteinatlas.org/) to determine the expression of these 11 metabolic proteins in the ccRCC tissues. The ALDH3B2 and FADS2 protein levels were significantly higher in the ccRCC tissues compared to the normal renal tissue (Figure 11C, 11H). Furthermore, NOS1, ALAD, ACADM, ITPKA, IMPDH1, SCD5, and CA4 levels were significantly reduced in the ccRCC tissues compared to the normal renal tissues (Figure 11A, 11B, 11D–11G, 11I). The expression of HK3 protein was similar in the ccRCC and normal renal tissues (Figure 11J).

## DISCUSSION

Cancer cells reprogram metabolism to gain survival advantage when competing with normal cells for limited nutrient resources. The ccRCC cells demonstrate the Warburg effect characterized by enhanced glucose uptake and glycolysis in normoxia conditions [13]. Furthermore, oncogenic mutations cause changes in glycolysis, fatty acid biosynthesis, and amino acid metabolism in the ccRCC tissues [14, 15]. However, the genes that drive these metabolic changes in ccRCC tissues are not clear. Moreover, the clinical significance of the metabolic genes in the diagnosis, treatment and prognosis of ccRCC patients is not well characterized.

In the current study, we identify 124 differentially expressed metabolic genes based on transcriptome data analysis of ccRCC and normal kidney tissues from the TCGA database. Functional enrichment analysis shows that these 124 differentially expressed metabolic genes are related to pathways such as small molecule catabolism, amino acid metabolism, fatty acid metabolism, ribonucleotide metabolism, nucleoside phosphate biosynthesis, oxidoreductase activity, mitochondrial matrix, iron ion binding, transferase activity, and glycosyl transfer activity. These pathways are involved in the metabolic reprogramming-related alterations of the biological functions of ccRCC cells. Warburg effect or aerobic glycolysis is the most well characterized metabolic reprogramming process in cancer cells, wherein the cancer cells rely on lactic acid fermentation for their energy needs regardless of oxygenation levels; this metabolic reprogramming helps cancer cells to thrive under nutrient-deprived conditions commonly encountered in the tumor microenvironment and in metastasized tissues [16]. Several studies have shown abnormal expression of glucose transporters, glycolytic enzymes and some key enzymes of the tricarboxylic acid cycle in the ccRCC cells [17–19]. The tryptophan metabolic pathway is also altered in ccRCC cells because it generates metabolites that promote tumor growth and induce immunosuppression, thereby reducing the efficacy of interferon-like immunotherapy [20, 21]. Fatty acid metabolism is also altered in the ccRCC cells [22]. Fatty acid metabolism is a complex process regulated by various enzymes, including acyl-CoA dehydrogenase, hydroxyacyl-CoA dehydrogenase and enoyl-CoA hydratase [23]. Elevated expression of fatty acid synthase is associated with tumor invasiveness and poor OS rates in ccRCC patients [24]. Furthermore, alterations in the tricarboxylic acid (TCA) cycle, electron transport chain (ETC), and glutamine and arginine metabolic pathways contribute to changes in the energy supply and intrinsic antioxidant properties of the ccRCC cells, thereby promoting tumor progression [19, 25]. Therefore, alterations in the expression of metabolic genes modulate several metabolic pathways involved in the synthesis and breakdown of amino acids, fatty acids, and nucleotides, mitochondrial oxidative phosphorylation, and other pathways. Thus, metabolic reprogramming alters the ATP and oxidative stress levels in the tumor cells, and also provides metabolic intermediates that serve to modulate immune cell infiltration in the tumor microenvironment, thereby influencing tumor growth and progression.

In addition, we constructed a PPI network for these differentially expressed genes and screened 10 hub genes including *GAPDH, POLR3B, NME1-NME2, ADCY10, ADCY7, POLR2F, NME1, ENTPD2, ADCY8, and ADCY3*. Among these genes, ADCY10, ADCY7, ADCY8, and ADCY3 are members of adenylate cyclase family, which catalyzes ATP generation of cAMP [26]. They have different reactions to upstream regulatory pathways and their distribution, and play an important role in tumorigenesis [27]. GAPDH has been found to play an important role in tumor cell survival, tumor angiogenesis, control of tumor cell gene expression, and post-transcriptional regulation of tumor cell mRNA [28]. And we found that ccRCC is associated with cellular amino acid metabolic process, generation of precursor metabolites and energy, organic acid biosynthetic process, nucleoside phosphate biosynthetic process, ribonucleotide metabolic process, and metabolic pathways through the PPI network module analysis.

Moreover, our analysis identified eleven prognosis-related metabolic genes, including *NOS1, ALAD, ALDH3B2, ACADM, ITPKA, IMPDH1, SCD5, FADS2, ACHE, CA4,* and *HK3*. NOS1 is a nitric oxide synthase that generates nitric oxide and plays an important role in the development, progression and metastasis of various tumors [29]. NOS1 also generates reactive oxygen species including superoxide ions (O_2_^−^) and hydrogen peroxide (H_2_O_2_) at low arginine concentrations [30]. The activity of NOS1 is closely related to protein tyrosine nitration [31]. Renaudin et al. demonstrated that NOS1 expression correlated with the pathological grade of renal tumors [32]. ALAD catalyses the second step of heme biosynthesis and is an endogenous inhibitor of 26S proteasome, which is a therapeutic target for several tumors [33–35]. *ALAD* gene variants are associated with the risk of genitourinary tumors [36]. ALDH3B2 is a member of the aldehyde dehydrogenase family that plays an important role in exogenous drug metabolism [37]. ALDH3B2 polymorphisms are related to the esophageal squamous cell carcinoma in the Chinese population [37]. ALDH3B2 also plays an important role in colorectal carcinogenesis [38, 39]. ACADM is an enzyme that catalyzes the initial step of mitochondrial fatty acid-oxidation pathway [40]. In this study, we demonstrate that ACADM is downregulated in ccRCC tissues. Huang et al. demonstrated that HIF-1 promotes various cancer progression by inhibiting fatty acid oxidation and acyl-CoA dehydrogenases [40]. Niu et al. showed that inhibition of ACDAM activity accelerated breast cancer progression in the mouse model [41]. Amino acid metabolizing enzymes play an important role in the evasion of tumor cells from immune surveillance [42]. IPTKA is a member of the inositol triphosphate kinase family that regulates actin dynamics and Ins (1, 4, 5) P3-mediated calcium signaling [43]. IPTKA is upregulated in several cancers, including those of the breast, lung, colon, liver, prostate and testis [43, 44]. Our study demonstrates that IPTKA mRNA levels are significantly increased in the ccRCC tissues. Guan et al. reported that high expression of IPTKA correlates with higher vascular infiltration and shorter survival time of liver cancer patients [45]. Liu et al. reported that ITPKA gene expression and the IPTKA subtypes correlate with the prognosis of ccRCC patients [46]. IMPDH is a rate-limiting enzyme in the biosynthesis pathway of guanosine triphosphate. It has two subtypes, IMPDH1 and IMPDH2, and is essential for DNA and RNA synthesis and signal transmission in all organisms [47]. In this study, we demonstrate that IMPDH1 mRNA levels are significantly increased in ccRCC tissues. Ruan et al. also reported that high IMPDH1 expression is associated with shorter overall survival and disease-free survival of RCC patients [47]. In a mouse model of non-small cell lung cancer (NSCLC), inhibition of IMPDH1 expression reduced the expression of RNA polymerase-I-dependent pre-ribosomal RNA expression, inhibited the growth of tumor cells, and improved the survival of NSCLC model mice treated with chemotherapy [48]. SCD proteins regulate the biosynthesis of cellular lipid fatty acids. SCD1 and SCD5 are the main subtypes in humans. The monounsaturated fatty acids produced by these enzymes promote the mobility of cell membranes and the growth rate of cancer cells. SCD1 is up-regulated in cancer cells and plays an important role in tumor progression [49]. Our study found that SCD5 mRNA levels were significantly down-regulated in ccRCC tissues. Bellenghi et al. reported that the expression of SCD5 was significantly higher in the primary melanoma cells, but significantly decreased in the metastatic melanoma cells; SCD5 overexpression in the melanoma mouse model significantly reduced the invasiveness of the melanoma cells [49]. Fatty acid desaturase 2 (FADS2) is an enzyme that catalyzes the biosynthesis of highly unsaturated fatty acids (HUFAs) [50]. Our study demonstrates that FADS2 mRNA expression is significantly up-regulated in the ccRCC tissues. Tian et al. showed that FADS2 was overexpressed in the colorectal cancer (CRC) tissues and promoted CRC cell proliferation [50]. The *ACHE* gene encodes the acetylcholinesterase, an enzyme that hydrolyzes acetylcholine, which is involved in signal transmission [51]. The gene structure and expression of ACHE is altered in various tumors [51, 52]. Motamed-Khorasani et al. reported that high acetylcholinesterase levels correlated with shorter overall survival in ovarian cancer patients [53]. Perry et al. demonstrated that *ACHE* gene variants were associated with the aggressiveness of human astrocytomas [54]. CA4 is a class of zinc-containing metalloenzymes that catalyze the reversible hydration of carbon dioxide and the dehydration of carbonic acid [55]. The down-regulation of CA4 cognate CAI or CAII is associated with colorectal carcinogenesis [56]. Hexokinase is the first step in catalyzing glycolysis. A recent study found that HK3 plays an important role in acute promyelocytic leukemia [57]. Pudova et al. reported that HK3 overexpression was associated with the epithelial-mesenchymal transition (EMT) of colorectal cancer cells [58]. Our study suggests that these eleven key metabolic genes may participate in the functional regulation of ccRCC by modulating metabolism.

We then used multivariate Cox proportional hazards regression analysis to establish a prognostic risk score model to predict the prognosis of patients with ccRCC. The ROC curve of the risk score model indicates moderate to good performance in predicting one-year OS (AUC= 0.810), three-year OS (AUC=0.738), and five-year OS (AUC=0.771). We then developed a nomogram based on the eleven metabolic genes to help doctors determine the prognosis of the ccRCC patients and decide on the therapeutic strategy. We showed that the prognostic risk score based on these eleven genes was an independent prognostic factor associated with OS. Kaplan-Meier survival curve analysis showed that low expression of *NOS1, ALAD, ACADM, SCD5*, and *CA4* genes and high expression of *ALDH3B2, ITPKA, IMPDH1, FADS2, ACHE*, and *HK3* genes was associated with shorter overall survival of ccRCC patients. These results demonstrate that the prognostic risk signature of these eleven metabolic genes can be used for determining the recurrence risk stratification, treatment outcomes, prognostic prediction. These genes may also serve as potential therapeutic targets.

In the past decade, advances in molecular science and the identification of new molecular biomarkers have shed newer insights into the biology of ccRCC. This has resulted in the development of new targeted therapies and cancer-related biomarkers, including, proliferation markers such as Ki-67, p53 and PTEN, hypoxia-inducible factor pathways, carbonic anhydrase IX, vascular endothelial growth factor (VEGF) and others [59]. Several studies have investigated the relationship between the somatic mutations, variations in gene methylation, differential gene expression, germline variations, and the status of immune biomarkers such as CD8 and PD-L1 with prognosis of ccRCC, and several different prognosis models have been proposed [60–62]. Brannon et al. identified two subtypes of ccRCC based on stratified consensus clustering of gene expression microarray data, and developed a 34-gene classifier for localized ccRCC [63]. Rini et al. analyzed the expression of 732 genes in 942 patients with stage I-III ccRCC, and selected eleven genes of interest including five reference genes to determine a continuous recurrence score [64]. Klatte et al. used tissue microarray technology to determine the correlation between the expressions of Ki-67, p53, VEGFR-1 and VEGF-D with ccRCC patient survival time, and constructed a prognostic model in combination with other clinical factors [65]. Recently, Zhao et al. constructed a prognostic model based on the expression status of three prognostic N6-methyladenosine genes [66]. In this study, we systematically analyzed the expression of metabolic genes that regulate metabolic reprogramming in ccRCC and developed a new prognostic model based on 11 metabolic genes.

This study has some limitations. Firstly, our study is based only on bio-omics data. However, we need to consider that analysis of different patient characteristics on different platforms can lead to patient heterogeneity. Secondly, the prognostic model construction and verification in our study was based on retrospective data analysis. Therefore, our findings need to be verified with a multicenter, large prospective cohort of ccRCC patients. Thirdly, variability in the clinical information from different datasets may reduce the statistical reliability and effectiveness of the Cox regression analysis. Finally, we used bioinformatics techniques to evaluate the diagnostic and prognostic prediction value of several key metabolic genes in ccRCC. However, the specific functions and mechanisms of these key metabolic genes in the growth and progression of ccRCC have not been well characterized, and hence, require further in-depth investigations.

In summary, we systematically studied the biological function and prognostic value of differentially expressed metabolic genes in ccRCC by a series of bioinformatics techniques. We also established a prognostic risk score model based on 11 metabolic genes, which proved to be an independent prognostic factor that can accurately predict the overall survival time of ccRCC patients. Our results will be of great significance in revealing the pathogenesis of ccRCC and developing new therapeutic targets or prognostic molecular markers.

## MATERIALS AND METHODS

### RNA-seq data analysis of ccRCC patients

We downloaded the transcriptome data consisting of 72 normal renal and 539 ccRCC tissue samples from The Cancer Genome Atlas database (TCGA, https://portal.gdc.cancer.gov/). We also downloaded the clinical data of all the ccRCC patients. We also obtained seventy metabolism-associated gene sets from the GSEA database (https://www.gsea-msigdb.org/gsea/msigdb/collections.jsp#C2). For data analysis, we first pre-processed the raw RNA-seq data with the edgeR package (http://www.bioconductor.org/packages/release/bioc/html/edgeR.html). This included averaging the genes with the same name, removing the genes with average expression of less than 1, and normalizing the expression data with the trimmed mean of M-values (TMM) normalization algorithm. We then identified the differentially expressed metabolic genes using |log_2_ FC| >1.0 and false discovery rate (FDR) <0.05 as the selection criteria.

### Functional enrichment analysis of the differentially expressed metabolic genes

We performed a comprehensive functional and pathway enrichment analysis of the differentially expressed metabolic genes in ccRCC using the WEB-based Gene Set Analysis Toolkit (WebGestalt, http://www.webgestalt.org/). This included identifying the GO terms for the cellular components, biological processes, and molecular functions, as well as the enriched KEGG signaling pathways in which the differentially expressed genes are enriched. GO terms and KEGG pathways with *P*<0.05 and FDR <0.05 were considered statistically significant.

### Construction of protein-protein interaction (PPI) networks and identification of key co-expression modules

The STRING database (https://string-db.org/) was used to identify the protein-protein interactions involving all the differentially expressed metabolic genes. Then, the PPI network was built using the Cytoscape 3.8.0 software (https://cytoscape.org/). The MCODE plug-in was used to select key modules based on the MCODE score and the node number. The cytoHubba plug-in was used to screen the hub genes according to the maximal clique centrality (MCC) algorithm. The GraphPad Prism 5.0 software was used to perform receiver operating characteristic (ROC) curve analyses of the hub genes to assess their ability to differentiate between normal and ccRCC tissues.

### Analysis of hub gene mutation frequency and copy number variation

The cBioPortal (https://www.cbioportal.org/) database was used to determine the gene mutations (missense mutations, gene amplifications, and deletions) and copy-number alterations of the hub genes. Then, the correlation between the hub gene mutations and survival time were determined by the survival module in the cBioPortal database.

### Identification of prognosis-associated metabolic genes

The caret R package was used to divide the ccRCC patients from TCGA cohort into training and test groups. Univariate Cox regression analysis was then performed for the differentially expressed metabolic genes in the training group using the survival R package. These genes were further screened using the LASSO regression analysis algorithm to identify the potential prognosis-associated metabolic genes, which were then subjected to multivariate Cox regression analysis to identify metabolic genes that can independently predict prognosis of ccRCC patients.

### Evaluation of the 11-metabolic gene signature-based prognostic model

The multivariate Cox proportional hazards regression model was established based on the selected metabolic genes to predict the prognosis of patients. The risk score of each patient was calculated according to the following formula:

$$Risk\;score=\sum_{i=1}^{n}Expi\beta i,$$

where, β is the regression coefficient of each gene and Exp is the expression value of each gene. We then divided the training group patients into high- and low-risk groups based on the median risk score. Then, the log-rank test was used to compare the differences in overall survival times between the two groups. Furthermore, we generated ROC curves using the survival ROC package to evaluate the model performance. The rms R package was used to construct a nomogram to predict survival probabilities. Finally, we used the test group and the E-MTAB-1980 dataset (https://www.ebi.ac.uk/arrayexpress/experiments/E-MTAB-1980/) to validate the predictive value of the prognostic model. In addition, we used the Kaplan-Meier plotter (https://kmplot.com/analysis/) online tool to verify the prognostic value of these 11 metabolic genes, and used The Human Protein Atlas (http://www.proteinatlas.org/) online database to detect the protein expression of these 11 metabolic genes.

## Supplementary Material

Supplementary Table 1

Supplementary Figure 1
