# Supplementary material for: An eleven metabolic gene signature-based prognostic model for clear cell renal cell carcinoma
Source: Aging (Albany NY). 2020 Nov 18;12(22):23165–86. doi: 10.18632/aging.104088 (PMC7746370; doi:10.18632/aging.104088)
Supplement: Supplementary Table 1 [file aging-12-104088-s001..pdf]

SUPPLEMENTARY TABLE

Supplementary Table 1. Univariate Cox regression analysis to identify prognosis metabolic genes in the TCGA.

Gene name	HR	*P* value	Gene name	HR	*P* value
ACADM	0.5802	4.01E-08	HMGCS2	0.8956	9.85E-05
ACHE	1.3247	7.16E-07	IMPDH1	2.1759	4.47E-09
ACLY	0.7799	0.0098	ITPKA	1.2809	4.60E-10
ADCY10	1.2619	0.0017	LRAT	1.1379	0.0222
ADH7	1.1916	0.0111	NOS1	0.8395	0.0033
ALAD	0.5415	1.51E-05	OXCT2	1.1932	0.0171
ALDH3B2	1.1657	0.0013	PCCA	0.5600	8.29E-08
ALDH6A1	0.6252	6.75E-08	PGK1	0.6002	0.0001
ALOX5	1.1594	0.0311	PLA2G2D	1.1194	0.0041
ATP4A	1.1907	0.0026	PLOD3	1.4620	0.0207
CA4	0.8238	3.01E-06	POLE2	1.5127	0.0002
CA6	1.2337	0.0164	POLR2F	1.2325	0.0220
CSAD	1.3518	0.0001	POLR3B	0.6935	0.0498
CYP4A11	0.9089	7.56E-05	PRODH	0.9227	0.0453
CYP4F3	1.1068	0.0070	RRM2	1.4365	9.25E-06
DEGS1	1.2887	0.0264	SCD5	0.7803	2.06E-05
FADS2	1.3121	0.0014	STX4	2.3725	1.03E-05
FMO4	0.7187	0.0068	SUCLG2	0.6215	0.0081
GAPDH	1.3166	0.0487	TAT	1.2076	0.0010
GK	0.8063	0.0257	TYMP	1.6270	5.93E-06
GPD1L	0.6178	0.0011	UGT2A3	0.9200	0.0045
GYS1	1.4391	0.0218	UGT8	0.7899	0.0003
HK3	1.5391	6.60E-06	VAMP1	1.609913351	1.23E-07
