# Supplementary material for: An eleven metabolic gene signature-based prognostic model for clear cell renal cell carcinoma
Source: Aging (Albany NY). 2020 Nov 18;12(22):23165–86. doi: 10.18632/aging.104088 (PMC7746370; doi:10.18632/aging.104088)
Supplement: Supplementary Figure 1 [file aging-12-104088-s002..pdf]

## SUPPLEMENTARY FIGURE

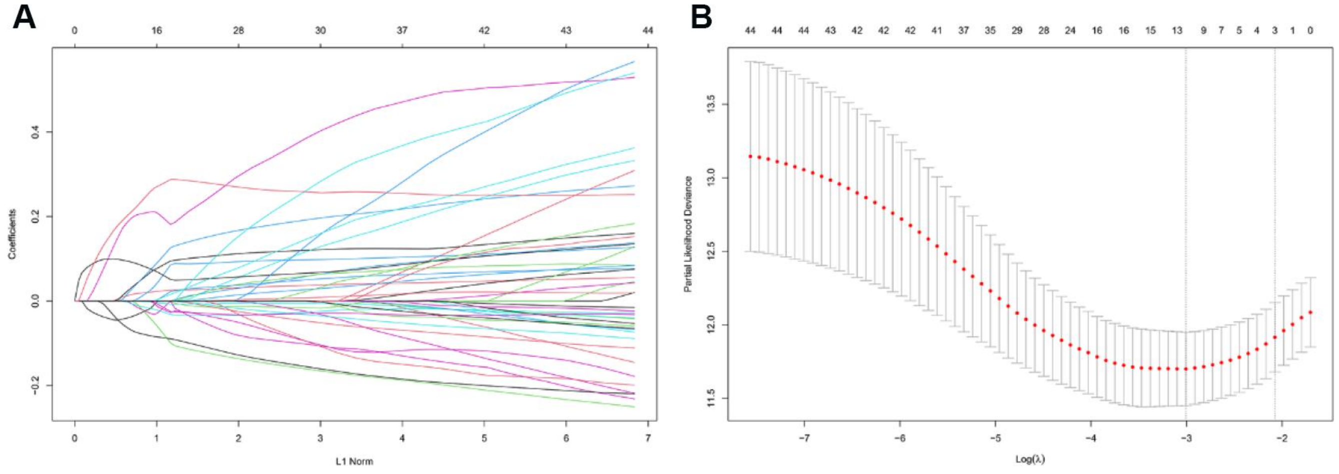


**Supplementary Figure 1. LASSO regression analysis for screening prognosis-related metabolic genes.** (**A**) Trajectories of model coefficients; (**B**) Cross validation fitting and performance evaluation of the model.
